# Service delivery redesign is a process, not a model of care

**DOI:** 10.1136/bmj-2022-071651

**Published:** 2023-03-13

**Authors:** Sanam Roder-DeWan, Supriya Madhavan, Savitha Subramanian, Kojo Nimako, Talib Lashari, Amith Nagaraj Bathula, Ramkumar Sathurappan, Sampath Kumar, Mickey Chopra

**Affiliations:** 1 World Bank, Washington, DC, USA; 2 Dartmouth Medical School, Hanover, NH, USA; 3 Bill and Melinda Gates Foundation, Seattle, Washington, USA; 4 World Bank Group, Ghana Country Office, Accra, Ghana; 5 Islamic Republic of Pakistan, Karachi, Sindh, Pakistan; 6 World Bank Group, India Country Office, Delhi, India; 7 Government of Meghalaya, Shillong, Meghalaya, India

## Abstract

**Sanam Roder-DeWan and colleagues** call for wider application of the principles of service delivery redesign to provide accessible, high quality services across healthcare

Achievements in expanding access to care will not result in better health outcomes without wider measures to improve quality of care. In 2018, the Lancet Global Health Commission on High Quality Health Systems proposed a fundamental shift from small scale quality improvement interventions towards more systems based solutions in low and middle income countries. Attention to improving quality at scale is growing, with substantial interest in one improvement approach recommended by the commission: service delivery redesign (SDR). 

SDR is the intentional reorganisation of a health system to improve equity, quality, and outcomes. To illustrate, an SDR programme intended to improve the quality of care for non-communicable diseases would start with a macro level analysis of the health system, identify where quality services have the highest potential of being delivered and where people prefer to receive these services, and then mobilise or build complementary interventions to make equitable access to quality services possible for everyone. For non-communicable diseases, this usually means shifting screening and management of uncomplicated disease to communities and homes. SDR leverages the organisation of a health system to rationalise services and make the right care available at the right level and at the right time. 

Unfortunately, in the years since the publication of the commission report, SDR has become nearly synonymous with one model of care for one set of services—that is, shifting childbirth to hospitals.^1^ Such a narrow definition assumes this is the correct model of care for each country or that one model can be seamlessly transplanted across settings. Although there is strong evidence and a salient ethical argument that everyone should have access to high quality, comprehensive emergency obstetric and newborn care within about 30 minutes of place of birth,^2^ equating SDR with hospital birth is erroneous, limiting, and potentially harmful. For example, many health systems that have increased use of hospitals for childbirth now struggle with high rates of caesarean deliveries that could hamper or reverse improvements in maternal and newborn health.^3^ ^4^ Reducing SDR to this single “travelling model”^5^ risks wasted resources and misses out on the potential of the method. In contrast, viewing SDR as a process that makes local expertise accessible has the potential to improve quality of care and outcomes in multiple settings and for many aspects of care.

## Service delivery redesign in practice

SDR is often misunderstood. Quality improvement interventions are not SDR unless they affect the organisation of a health system. Similarly, reorganisation programmes—such as the development of networks of care^6^—may not be SDR if reorganisation is not intended to equitably overcome “quality ceilings” at a given level of the health system. 

A variety of SDR models are being developed and tested in maternal and newborn health. The redesign process has allowed local expertise and innovation to point to solutions for a particular health problem that work in a particular setting. 

In Meghalaya State, India, district leaders are developing “hub and spoke” models that use freestanding birth centres to expand high quality care within 30 minutes of comprehensive emergency obstetric and newborn care for indigenous populations living in remote villages. The state is partnering with Tamil Nadu to provide specialised training to government doctors serving these communities, providing mobility support for health outreach staff and pregnant women, setting up maternity waiting homes with the help of local civil society, expanding autonomy for medical officers in their jurisdictions, and working with the World Bank to improve roads between hub facilities and their birthing satellites.^7^

The government of Kakamega County, Kenya, opted to focus its SDR programme on increasing the number of women who give birth in hospitals. Improvements in capacity and quality in hospitals have been complemented by transportation interventions, participatory work in communities to raise demand for quality services, and improvements in antenatal and postnatal care.^8^

In Tanzania, access to high quality definitive care for childbirth was achieved in Kigoma region by upgrading primary care centres to deliver emergency obstetric and neonatal care services, building on the existing decentralised birthing model. Improvements in facilities together with training and support for staff substantially improved birth outcomes.^9^

In some of the countries with the highest maternal and newborn mortality, such as Chad and Niger, SDR is being used to solve extreme geographic and health system performance challenges and prioritise scarce resources to improve health outcomes and engender trust in the health system. To complement important public health and family planning programmes, SDR in these countries focuses on strengthening district hospitals and implementing interventions to improve access to care to maximise use at or close to these facilities. Geospatial analytics have helped identify health system infrastructure in areas of extreme poverty so that interventions are appropriately targeted.

For many services, such as screening and management of non-communicable diseases, the contextual and system factors that enable high quality care are less clear than for maternal and newborn health. For example, the range of services that could be delivered at higher quality if shifted from hospitals to the community is likely to be both highly variable and context specific. The same is true for the changes required in any given health system to preserve continuity of care across different levels. Middle income countries in particular are exploring redesign within the context of primary healthcare reform. Côte d’Ivoire and Ghana, for example, are planning national reforms to reorganise their healthcare delivery systems into networks of care to improve quality of care, starting with policy change and legal commitments to equitably expand high quality care to all.^10^ ^11^ Ghana is using implementation research to learn from the scale-up of networks and from variation in network design across different districts. 

The government of Eswatini, with the support of the World Bank, redesigned services during the height of the covid-19 pandemic in response to community preference. Hypertension and diabetes care were decentralised to communities while reserving hospitals for the influx of people with acute respiratory illness.^12^ A pilot of this programme showed improvements in blood pressure control and processes of care. 

As these examples show, there is no single model of SDR. There is, however, a process for developing, implementing, and evaluating SDR models of care.

## Model for consultative, evidence based change

**Fig 1:**
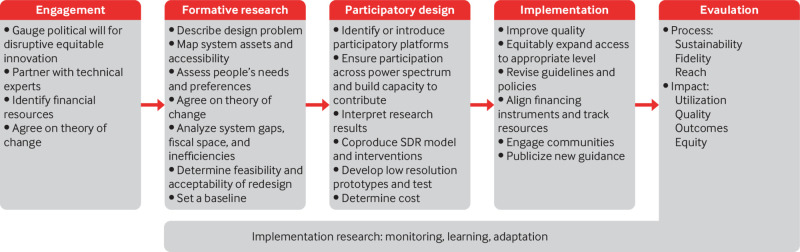
Five phases of service delivery redesign

The SDR process occurs across five, not strictly linear, phases beginning with an engagement period (fig 1). Deliberate redesign requires establishing political support for change along with administrative capacity and country commitment to assign budgets. Successful lasting change requires tightly matched incentives and interests, and consultative processes. Creating a multistakeholder coalition to steward the redesign process reduces the risk that reforms will slow or stall because of changing leadership or other geopolitical calamities. Support for change from financial leadership is also essential to ensure that the process is adequately funded and ongoing budgets are guaranteed.^13^

If the engagement phase suggests that the redesign process has sufficient support, mixed methods formative research is used to fill gaps in data on quality and peoples’ preferences for care, answer key implementation questions (ie, feasibility, acceptability), and set a baseline against which to measure the programme.^8^ Formative research also seeks to find major sources of inefficiency in the health system and quantify the possible cost savings from the greater efficiency and health improvements expected to follow from SDR.

During the participatory design phase, novel models of care and innovative solutions that support the redesign are developed. For example, in the SDR process in Kakamega County, mothers expressed dissatisfaction with queue management at antenatal clinics during the participatory design workshops. This led to the co-development of a ticketing system whereby mothers receive numbers when they enter the clinic, to allow them to know their place in the queue. Mothers have reported feeling empowered and fairly treated, and health workers describe fewer disagreements with clients regarding queueing with the new ticketing system. 

The participatory design phase should build on existing platforms for community participation, with particular attention to including less powerful members of society and those living in remote and underserved regions to ensure that the specific challenges they face are taken into account.^14^ Continued active participation of the financing leadership is also needed at this stage to ensure the redesign process is properly funded.

The key components of the implementation phase of SDR for maternal and newborn health have been described elsewhere,^2^ and are similar across other healthcare services. Facilities and networks are strengthened and expanded to prepare for shifts in usage and service delivery. Partnerships are developed with communities, financing instruments are aligned, and policies and guidelines are updated. Monitoring, learning, and implementation research are necessary throughout the process to identify and respond to unintended negative consequences, overcome challenges, and adapt approaches, including on the financial aspects of redesign.

## Shared principles to guide future redesign

Although the most appropriate approach to redesign a model of care will differ for each health system, four key principles can guide SDR efforts to improve quality across settings.

*Start with the hardest to reach populations*—To expand equitable access to high quality care and commit to universal health coverage we need to move beyond documenting inequities and find solutions to reach underserved populations.^15^ For example, Pakistan is using children who have not received any vaccinations as a proxy for access to poor quality care and then incorporating this information into developing criteria for an SDR programme designed to rebuild after the 2022 floods. Following the logic of targeted or progressive universalism, if the health system is robust enough to meet the needs of this last mile population, less underserved populations will also benefit.^16^

*Centralise services for complex and life threatening conditions—*Management of complex conditions or conditions that can rapidly deteriorate into life threatening clinical challenges requires considerable expertise. Concentrating highly skilled care for, say, psychiatric emergencies in fewer facilities allows providers with higher volumes to maintain skills for managing these conditions. Improvement efforts can also be better targeted at the types of cases seen and the resources available at one level of care.

*Localise care of non-communicable and minor illnesses—*Care for people with ongoing and ambulatory conditions, especially those that require frequent visits with providers, is best delivered close to communities. This principle is a basic tenet of primary healthcare as outlined in the Declaration of Alma Ata and facilitates delivery of care which is contextually appropriate and informed by the socioeconomic realities of peoples’ lives. For example, a provider who lives and works in the same community as a patient with type 2 diabetes will have knowledge of local food sources and customs and be better able to help that patient make realistic but healthy dietary changes.

*Use evidence based models of care—*New models of care introduced as part of SDR must be evidence based and informed by peoples’ healthcare needs and preferences. There must be a “match” between these needs and preferences and proposed models. For example, a decentralised model of comprehensive emergency obstetric and newborn care, such as in the Kigoma region of Tanzania,^9^ may not work where people are bypassing local facilities and already seek obstetric care in hospitals. When community preferences are not clinically advised—eg, giving birth at a home remote from a centre with emergency management facilities—providers should have the capacity to communicate risk and share decision making. 

The desire for a disruptive strategy to reduce stagnant maternal and perinatal mortality rates and the need for a systemic approach to handle the rise in non‑communicable diseases in low and middle income countries have led many to mobilise national, international, and private sector resources to explore and test programmes grounded in the principles of SDR. Efforts to build more resilient and responsive health systems after the service disruptions of covid-19 have also fed into enthusiasm around SDR. The process of examining, and considering changes in, health system design entailed in SDR means these efforts are inherently context specific. When based on shared, underlying principles, including equity and centring patients’ needs and preferences, the process of SDR can unlock innovative solutions to improve quality.

**Key messages**

Service delivery redesign has become synonymous with one model of care for one condition.
Service delivery redesign is a process that allows development of innovative solutions that are tailored to specific needs
Government support and community input from the start help ensure improvement in quality of care

## Notes

Provenance and peer review: Commissioned; externally peer reviewed.

This article is part of a collection proposed by the World Health Organization and the World Bank and commissioned by *The BMJ*. *The BMJ* peer reviewed, edited, and made the decision to publish these articles. Article handling fees are funded by the Bill and Melinda Gates Foundation. Jennifer Rasanathan, Juan Franco, and Emma Veitch edited this collection for *The BMJ*. Regina Kamoga was the patient editor.
